# Turbo autoencoders for the DNA data storage channel with Autoturbo-DNA

**DOI:** 10.1016/j.isci.2024.109575

**Published:** 2024-03-27

**Authors:** Marius Welzel, Hagen Dreßler, Dominik Heider

**Affiliations:** 1 Department of Mathematics and Computer Science, University of Marburg, 35043 Marburg, Hesse, Germany; 2 Department of Sustainable Systems Engineering, University of Freiburg, Fahnenbergplatz, 79085 Freiburg im Breisgau, Baden-Württemberg, Germany

**Keywords:** Biotechnology, Devices

## Abstract

DNA, with its high storage density and long-term stability, is a potential candidate for a next-generation storage device. The DNA data storage channel, composed of synthesis, amplification, storage, and sequencing, exhibits error probabilities and error profiles specific to the components of the channel. Here, we present Autoturbo-DNA, a PyTorch framework for training error-correcting, overcomplete autoencoders specifically tailored for the DNA data storage channel. It allows training different architecture combinations and using a wide variety of channel component models for noise generation during training. It further supports training the encoder to generate DNA sequences that adhere to user-defined constraints. Autoturbo-DNA exhibits error-correction capabilities close to non-neural-network state-of-the-art error correction and constrained codes for DNA data storage. Our results indicate that neural-network-based codes can be a viable alternative to traditionally designed codes for the DNA data storage channel.

## Highlights

• High error-correction performance
• Can be trained to adhere to user-defined constraints
• Supports custom DNA channel models
• High customizability

## Graphical abstract

**Figure:**
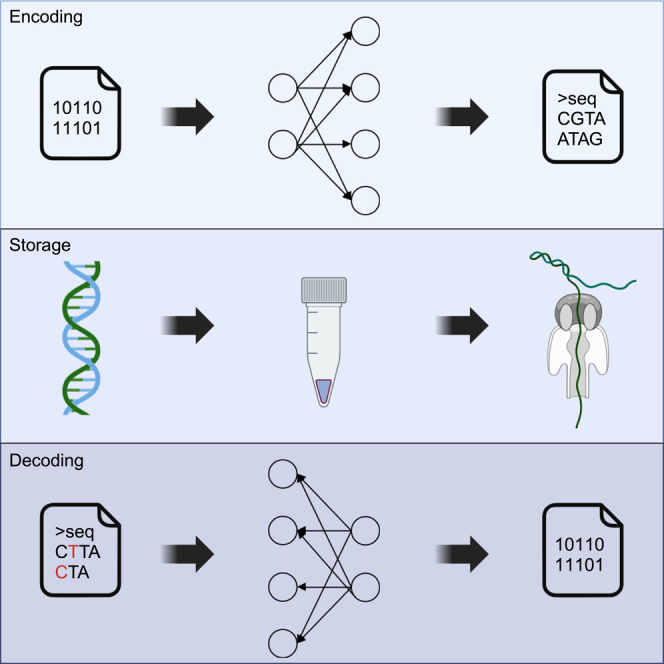

## Introduction

The exponential increase in data generation^1^ leads to an increasing demand for data storage solutions with a high storage density and long-term stability. The global demand for data storage is estimated to reach 175 Zettabyte (ZB) in 2025.^2^ DNA, with a storage density of around $10^{18}\frac{bytes}{mm^{3}}$, and a potential lifetime of hundreds of years,^1^ is one potential candidate for such a next-generation storage device. Utilizing DNA as a means to store data is an active field of research, with many advances that were reported in recent years.^3^^,^^4^^,^^5^^,^^6^^,^^7^^,^^8^^,^^9^^,^^10^ One important aspect of DNA as a data storage device is the error types that are typically observed in DNA: substitutions, which involve the change from one base to another, insertions of one or more bases into the DNA strand, which lead to a positional shift of all following bases to the right in the DNA strand, and deletions of bases that lead to all following bases being shifted to the left.^11^ The positional change of bases in a DNA strand increases the decoding complexity, and many different strategies have been developed for the decoding of sequences that contain indel (i.e., insertion or deletion) errors. One effective strategy involves encoding multiple copies of the same data block into several encoded blocks. This redundancy allows for error compensation. If one block has indel errors, another block with the same information can be used as a backup, as shown in studies by Schwarz and Freisleben^5^ and Erlich and Zielinski.^12^ Another approach presented in a study by Welzel et al.^13^ is maximum likelihood tree decoding. This approach utilizes the periodic insertion of synchronization markers for decoding data, allowing the decoder to identify positional shifts that occur in the presence of indel errors. A further approach is the exploitation of sequencing depth by using a form of majority voting for sequencing reads, which allows compensation for indels that occurred during sequencing.^14^ The DNA data storage channel consists of multiple steps, the writing of data into DNA (synthesis), the amplification of the synthesis product using PCR, the storage process itself, and the reading of the DNA back into a digital format (sequencing). Each component, including the various options for a component (for example, different sequencing machines), exhibits unique error profiles and error patterns.^15^ Incorporation of this knowledge into the design process of coding schemes for DNA data storage could potentially lead to more robust codes. A further consideration when designing codes for DNA data storage is constraint adherence. Some DNA data storage channel components exhibit increased error probabilities for some sequence patterns. For instance, in a DNA sequence, the content of guanine and cytosine (GC) should generally be in balance with the content of adenine and thymine. Sequences with a GC content that deviates substantially from 50% can lead to synthesis failures, unstable DNA, as well as sequencing errors.^4^ Similarly, large chains of the same base in a sequence (so-called homopolymers) can also lead to sequencing errors. The encoded DNA sequences should also be free of certain motifs specific to the synthesis or storage processes; for example, encoded sequences should not contain any restriction motifs used in the synthesis process or motifs with biological relevance if the encoded data are stored *in vivo*.^4^ Further constraints, such as the occurrence of short repetitive sequences (k-mers) or the probability of secondary structure formation of the encoded sequences, must also be considered in some cases.^15^ One possible way to incorporate sequence constraints, error profiles, and error patterns into a coding system that can be flexibly adjusted to different combinations of synthesis, PCR, storage, and sequencing would be to leverage the learning ability of neural networks (NN). Jiang et al.^16^ presented an end-to-end autoencoder coding system, TurboAE, that is solely based on NN but with a decoder that is arranged similarly to turbo codes.^17^ The authors showed that for some non-canonical channels, TurboAE outperforms state-of-the-art codes. Given the complexity of the DNA data storage channel, NN-based coding systems like TurboAE can learn the peculiarities of the DNA storage channel while being able to be fine-tuned on specific setups of synthesis, sequencing, and storage methods and PCR polymerases, which could lead to improvements regarding the error correction performance and execution time required for the en- and decoding processes. Another potential advantage of such coding systems is that they perform well for short and moderate block lengths.^16^ For DNA, it is practical and efficient to have shorter strands, as the probability of errors during synthesis and sequencing increase with longer strand lengths.^18^^,^^19^ These strand lengths are typically between 300 and 1,000 base pairs for current sequencing technologies, while shorter fragment sizes are cheaper to synthesize on a dollar-per-base basis.^18^ However, TurboAE requires that the input of the decoder is of a fixed length. Given that the DNA data storage channel can lead to indel errors that change the length of the sequence, TurboAE cannot be used for the DNA data storage channel without adjustments.

While DNA sequencing technologies have made tremendous progress in the last decades,^20^ and the amplification of DNA utilizing PCR being comparatively cheap, the high costs and slow throughput of DNA synthesis^18^ is a major bottleneck for large scale adoption of DNA as a storage device. The development of fast and cheaper synthesis technologies is therefore required for the adoption of DNA data storage.^1^ For a coding solution to be usable for economically viable DNA data storage, it has to be flexible so that it can be used with new synthesis technologies, and the, as of yet, unknown, error rates and error profiles of such new technologies.

Here, we present Autoturbo-DNA, an end-to-end autoencoder framework that combines the TurboAE principles with an additional pre-processing decoder, DNA data storage channel simulation, and constraint adherence check. Autoturbo-DNA supports various NN architectures for its components, which can be mixed and matched using a configuration file, combined with user-friendly adjustment of the DNA data storage channel and constraint adherence parameters. Autoturbo-DNA does not only allow the correction of errors common in DNA data storage, including indels, but can also be trained to generate sequences that adhere to various constraints. Furthermore, Autoturbo-DNA supports the configuration of the channel simulator using configuration files generated by the Mosla error simulator (MESA),^15^ allowing users to generate complex error profiles using a click-and-drag interface. This cross-compatibility enables Autoturbo-DNA to be trained on error profiles of new technologies as soon as they become available.

## Results

### Architecture overview

**Figure 1:**
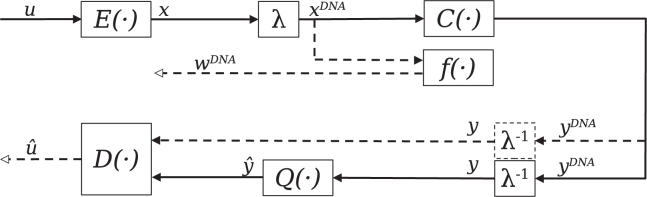
Overview of the main components of Autoturbo-DNA: A given binary input *u* is encoded using the encoder network E(·) and subsequently mapped to a DNA sequence by the mapping function λ The output of the mapping function $x^{DNA}$ is then modified by the channel simulator C(·) and optional evaluated for constraint adherence by the function f(·). The output score $w^{DNA}$ of the evaluation function can be used as an additional loss metric for the encoder. The inverse of the mapping function translates the DNA sequence back into a binary representation *y*. Depending on the training stage and chosen configuration, the channel output $y^{DNA}$ is either directly decoded by the decoder function D(·) or first transcoded using the indel reduction component Q(·), and the resulting sequence $\hat{y}$ is decoded by the decoder function, producing the binary output sequence $\hat{u}$.

**Figure 2:**
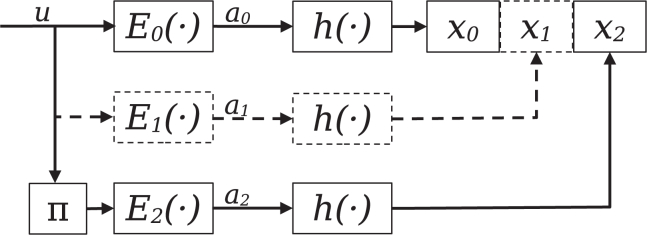
Schematic illustration of the encoder: It consists of two, with an optional third, networks $E_{0-2}\left(\cdot \right)$ The input data are copied and sent through each encoder network separately. For the encoder $E_{2}\left(\cdot \right)$, the input data are first interleaved using the interleaving function π before being encoded. Each encoder output is normalized and binarized before being concatenated into the encoded binary sequence *x*.

**Figure 3:**
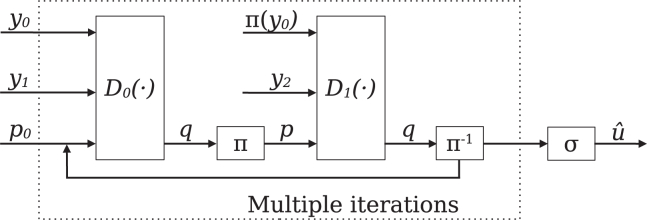
Schematic illustration of the decoder, shown for rate $\frac{1}{3}$ The input sequence *y* is split into the subsequences $y_{0}$, $y_{1}$ and $y_{2}$, which correspond to the encoded subsequences $x_{0}$, $x_{1}$ and $x_{2}$, respectively. $y_{0}$ and $y_{1}$, together with a prior $p_{0}$, are used as the input for the first decoder network $D_{0}\left(\cdot \right)$. The output posterior *q* and $y_{0}$ are subsequently interleaved and serve, together with $y_{2}$, as the input for the second decoder $D_{1}\left(\cdot \right)$. The output of the second decoder is deinterleaved by the inverse of the interleaving function, $\pi^{-1}$, and is used as updated prior for the first decoder. This process will be repeated until a user-defined amount of iterations is reached. The final, deinterleaved output of the second decoder will then be used to generate the output sequence $\hat{u}$ by a sigmoid activation function σ.

The autoencoder architecture of Autoturbo-DNA consists of three main components, summarized in Figure 1. The encoder E(·), shown in Figure 2 is composed of multiple NN that take as input a bit sequence and output two (optionally, three) encoded sequences. The indel reduction component Q(·) uses the channel output sequences of varying lengths as input and returns fixed-size sequences that serve as input for the decoder D(·), shown in Figure 3, that leverages Turbo code principles^17^ to iteratively improve predictions of the encoded sequence between two NN.

To showcase the versatility and flexibility of Autoturbo-DNA, we have trained models with different combinations of encoder architectures, transcoder architectures, and decoder architectures. This approach aims to isolate the effects of different encoder-decoder combinations on the overall performance and outcomes, providing an indicative illustration of the potential usage of the framework. To achieve this, we have set a fixed set of hyperparameters across all trials to give a snapshot of the framework’s functionality under constant conditions. The hyperparameters that deviate from the default values are listed in Table S6. The error probabilities used are a combination of column-synthesized oligos utilizing the ErrASE error correction, Illumina single-end sequencing, PCR amplification for 30 cycles using the TAQ polymerase, and storage in E.coli for 24 months. This combination of error sources would lead to a total error rate of 0.33%. The error rate was increased while keeping the distribution of error types and patterns, using the amplifier to a total error rate of 6.8%, which is closer to the error rate of other DNA data storage codes used for *in-vitro* storage that include constraint adherence.^13^ To compare different architecture and hyperparameter combinations, the reconstruction accuracy is utilized. This accuracy, for a single binary vector, is defined as the number of elements between the input vector $u=\left[u_{1},u_{2},u_{3},\ldots ,u_{n}\right]$ and the output vector $\hat{u}=\left[{\hat{u}}_{1},{\hat{u}}_{2},{\hat{u}}_{3},\ldots ,{\hat{u}}_{n}\right]$:

(Equation 1) $$\text{Rec}.\;\text{accuracy}=\frac{\sum_{i=1}^{n}\left\{ \begin{array}{cc} 1 & \text{if}\;u_{i}={\hat{u}}_{i}\\ 0 & \text{otherwise} \end{array}\right.}{n}$$

### Architecture comparison

The different neural network architectures supported by Autoturbo-DNA were compared in regards to their error-correction performance as encoder, transcoder, or decoder architecture. For the evaluations, we tested combinations of CNN, RNN, VAE, and Transformer-encoder for the encoder, CNN, RNN, and ResNet for the transcoder, and CNN, RNN, ResNet, and Transformer-encoder for the decoder. Each combination was trained for 400 epochs with an otherwise fixed set of hyperparameters. The results, separated by each part of the codec and by the different architectures, are shown in Figure S2 as boxplots, with the mean and median reconstruction accuracy for the different combinations, as well as outliers, marked. The results indicate that models utilizing either CNNs, VAEs, or RNNs as encoder architecture perform similarly well for the used hyperparameters and evaluation metrics. At the same time, a transformer-encoder has the lowest mean and median from all evaluated encoder architectures. ResNets as transcoder architectures have the highest mean and median and the smallest interquartile range for the evaluated hyperparameters and evaluation metrics. RNNs as transcoder architecture led to a median accuracy that is slightly higher than the median accuracy of CNNs. However, RNNs have a lower mean and a more extensive interquartile range than CNNs and RNNs. For the decoder architectures, CNNs had the highest mean and median accuracy and the smallest interquartile range. ResNets had a median accuracy close to CNNs but with a larger interquartile range, lower mean accuracy, and fewer outliers. Transformer-based encoders and RNNs as decoder architectures performed similarly with the chosen evaluation metrics and hyperparameters.

For further evaluations, we focused on VAE and CNN architectures for the encoder, CNN architectures for the decoder, and ResNets for the transcoder.

### Block length comparison

**Figure 4:**
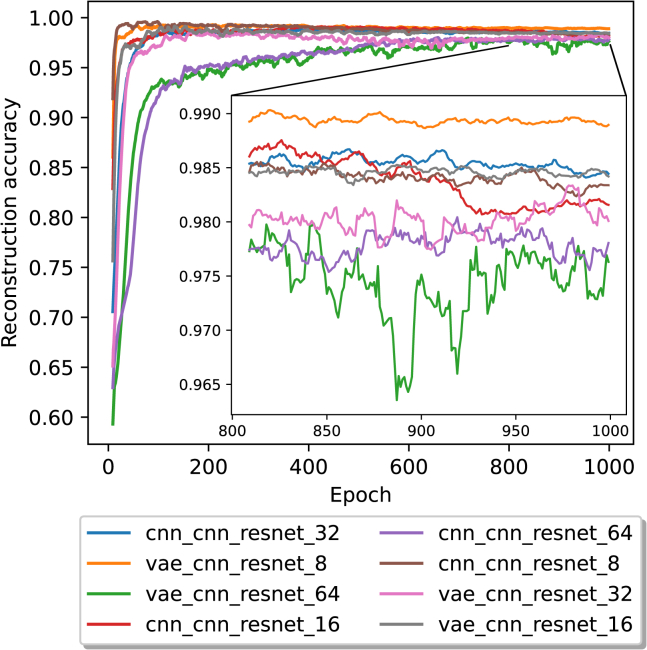
The average reconstruction accuracy in a 10 epoch rolling window for 1,000 epochs For visual clarity, the last 200 epochs are magnified. The legend labels are structured in the form of encoder, decoder, transcoder, and block size.

To evaluate the influence of the block length on the training time and reconstruction accuracy, we used the same set of parameters described in the architecture overview section. However, we increased the number of epochs to 1,000 to ensure the models have sufficient time to learn and extract features from the more complex, longer sequences, thereby effectively capturing their underlying patterns and structures. We used block lengths of 3·8,3·16,3·32, and 3·64 bits. The results of the reconstruction accuracy over time, with a rolling average over ten epochs, are shown in Figure 4.

With shorter block lengths, the reconstruction accuracy increased in fewer epochs than with larger block lengths and also reached higher peaks. With a block length of 3·8 bits, a model using a CNN encoder reached a peak reconstruction accuracy of 0.99744 after 90 epochs. In comparison, the VAE encoder model reached a peak of 0.99576 after 170 epochs. The CNN encoder-based model showed a clear downtrend in reconstruction accuracy over time (as shown in the supplement). With a block length of 3·16 bits, a CNN encoder model reached a peak reconstruction accuracy of 0.99318 after 438 epochs, and the VAE encoder model reached a peak reconstruction accuracy of 0.99455 after 148 epochs. With this block length, CNN encoder-based models also showed a downtrend in reconstruction accuracy over time. For a block length of 3·32 bits, the CNN encoder model peaked at a reconstruction accuracy of 0.99021 after 105 epochs, followed by a more stable progression than the shorter block lengths. The VAE encoder model reached a peak of 0.9901 reconstruction accuracy after 161 epochs. In contrast, with a block length of 3·64 bits, the CNN encoder model reached its peak of 0.984 reconstruction accuracy after 604 epochs, and the VAE encoder model reached its peak of 0.98452 after 937 epochs. The top-performing model, which achieved a final error rate of 0.9% after 1,000 epochs, outperformed the least effective model, with its final error rate of 2.536% after the same duration, by approximately 64.51%.

### Impact of latent redundancy

**Figure 5:**
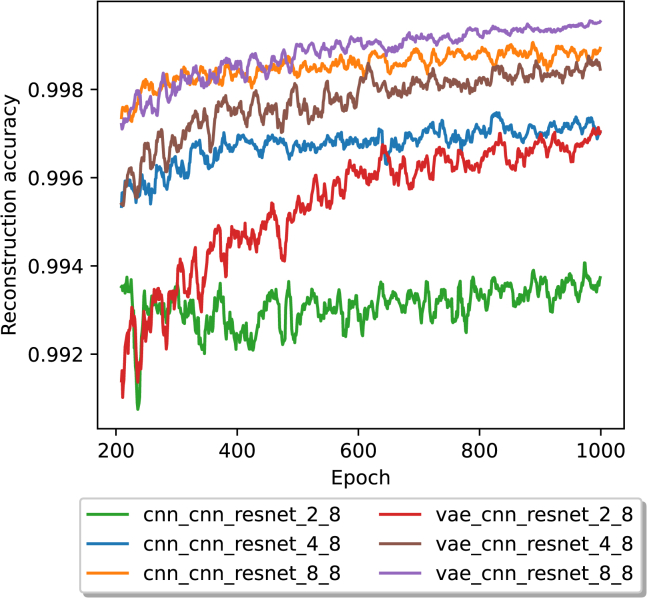
The average reconstruction accuracy in a 10 epoch rolling window for 1,000 epochs, beginning with epoch 200 and with a block size of 3·8 The legend labels are structured in the form of encoder, decoder, transcoder, latent redundancy, and block size.

Besides supporting the rates $\frac{1}{2}$ and $\frac{1}{3}$ by changing the number of encoded blocks that are generated per block of input data, Autoturbo-DNA also supports more fine-grading adjustments of the code rate, by increasing the number of units of the encoder layers, leading to an increase in the size of the latent representation. To analyze the impact of an increase in latent representation size, the same hyperparameters as before were used, but with the latent redundancy hyperparameter set to either 2 bits, 4 bits, or 8 bits and with a block length of either 3·8 or 3·16 bits. The results of the average reconstruction accuracy, from epoch 200 on, and for a block length of 3·8 bits are shown in Figure 5.

In contrast to the results without latent redundancy, no model showed signs of a downtrend. Each model had a higher reconstruction accuracy than any model without latent redundancy. For the CNN model with a latent redundancy of 2 bits, the reconstruction accuracy was 0.99442, while for the VAE model with the same amount of redundancy, the accuracy was 0.99725. For a latent redundancy of 4 bits, the CNN model had an accuracy of 0.99683, while the VAE model had an accuracy of 0.99817. For an 8-bit latent redundancy, the CNN model had an accuracy of 0.99899, and the VAE model had an accuracy of 0.99957. For each latent redundancy value, the accuracies of the models utilizing a VAE-based encoder are higher than those based on a CNN encoder. For all tested models and latent redundancy values, the slopes of the last 200 epochs are slightly positive, as shown in Table S7.

With a block length of 3·16, the reconstruction accuracy was lower for each encoder architecture and latent redundancy combination than the same combinations with a block size of 3·8. For the models using a CNN encoder, the accuracies were as follows: 0.99025 for a latent redundancy of 2 bits, 0.99272 accuracy for a latent redundancy of 4 bits, and an accuracy of 0.99545 for a latent redundancy of 8 bits. Utilizing a VAE-based encoder, the accuracies were 0.98605 for a latent redundancy of 2 bits, 0.98886 for a latent redundancy of 4 bits, and 0.99377 for a latent redundancy of 8 bits. In contrast to the evaluations where a block size of 3·8 was used, with a block size of 3·16, a CNN-based encoder leads to a higher accuracy score for all tested latent redundancies. Except for the CNN-based encoder utilizing a latent redundancy of 4 bits, all models had a slight positive slope for the last 200 epochs, as shown in Table S8.

In terms of error-correction performance, with the tested hyperparameters and training time, Autoturbo-DNA does not quite reach the error-correction performance of manually designed state-of-the-art codes like HEDGES^21^ or DNA-Aeon. With an error rate of 7.2%, HEDGES achieves a reconstruction accuracy of 1.0 with a code rate of 0.21, while DNA-Aeon is able to achieve a reconstruction accuracy of 1.0 with a code rate of 0.25, as shown in a study by Welzel et al.^13^ Autoturbo-DNA, on the other hand, achieves a reconstruction accuracy between 0.975 with a block size of 3·64, a VAE encoder, ResNet transcoder, and CNN decoder, at a code rate of $0.\bar{3}$, and a reconstruction accuracy of 0.99957, with a block size of 8, a VAE encoder, a ResNet transcoder, a CNN decoder and a latent redundancy of 8, with a code rate of $0.1\bar{6}$. To test the average run time per sequence, we encoded a 4.8 kilobyte text file. Tested on a ThinkPad E495 in central processing unit (CPU) only mode, with a Ryzen 5 2.1 GHz processor, Autoturbo-DNA required 2.7 s to encode the file into 697 strands of 96 bases each, or 0.004 s per strand. HEDGES required just 0.31 s to encode the file into 765 strands of 96 bases each, or 0.0004 s per strand. DNA-Aeon required 2.36 s to encode the file into 300 strands of 98 bases each, or 0.0079 s per strand. To decode the file, Autoturbo-DNA required 12.93 s, or 0.019 s per strand. HEDGES required 3.12 s to decode the data, or 0.004 s per strand. DNA-Aeon required 4.61 s, or 0.015 s per strand.

### Fine-tuning for constraint adherence

To train the encoder to generate DNA sequences that adhere to constraints, we have generated a configuration file using MESA that associates a 100% error probability to a sequence if it contains a homopolymer longer than three bases or if the GC content of the sequence is not between 40% and 60%. These constraints were chosen for comparability to other studies.^13^ The stability score $w^{DNA}$, which is used as an additional metric to train the encoder in addition to the reconstruction accuracy, is defined as:

(Equation 2) $$Stability\;score\;\left(w^{DNA}\right)=1-\frac{1}{n}\sum_{i=1}^{n}x_{i}$$

with $x_{i}$ being the error probability of the *i*th encoded block of batch size *n*. The error probability is the user-defined probability of an error occurring in the storage channel due to constraint breaches, i.e., GC content that is not in the range set by the user or homopolymer chains longer than desired. Training the models from the beginning using the stability score as an additional training metric for the encoder has only a slight impact on the stability score and reconstruction accuracy of the models, as shown in Figures S3 and S4.

**Figure 6:**
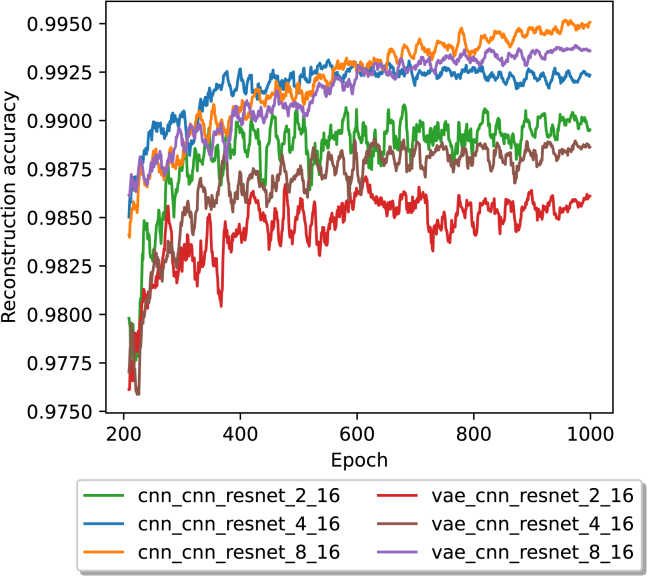
The average reconstruction accuracy in a 10 epoch rolling window for 1000 epochs, beginning with epoch 200 and with a block size of 3·16 The legend labels are structured in the form of encoder, decoder, transcoder, latent redundancy, and block size.

**Figure 7:**
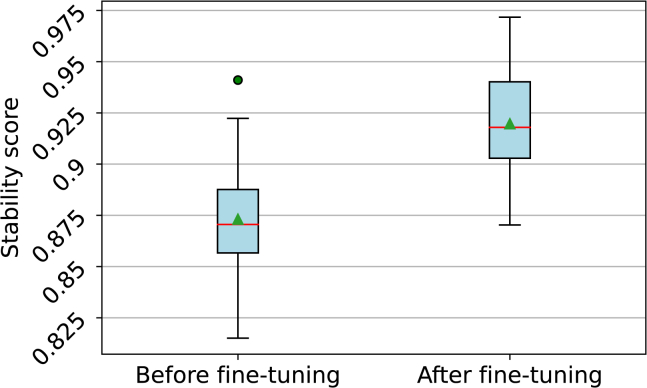
Boxplot of the stability score of models trained before (left) and after (right) fine-tuning for 100 epochs using the stability score as a training metric The models were further trained with either 2, 4, or 8 bits of latent redundancy and a block size of 8 or 16 bits. A red line represents the median, a green triangle represents the mean, and the outliers are represented by green dots. The difference in means between the two groups is statisticlly significant (t-statistic: 2.91, *p* value: 0.008).

Instead of training the models from the beginning using the additional constraint adherence training, we also fine-tuned the models that were trained with different amounts of latent redundancy (see Figure 6). The fine-tuning was carried out and led to a significant (t-statistic: 2.91, *p* value: 0.008) difference in the means between the two groups, as shown in Figure 7 and over the complete course of the training in the Figure S5. After fine-tuning, the mean and median of the reconstruction accuracies were slightly lower than before, as shown in Figure S6. The difference in means was, however, not statistically significant, with a t-statistic of −0.932 and a *p* value of 0.362.

## Discussion

Autoturbo-DNA is a feature-rich framework for training encoder-transcoder-decoder models for DNA data storage. The framework supports training using a DNA data storage channel simulator with a wide variety of options, all based on literature error rates and patterns of the individual DNA storage channel components. Utilizing the TurboAE structure first presented by Jiang et al.,^16^ combined with a transcoder to account for insertion and deletion errors, allows Autoturbo-DNA to reach reconstruction performance close to single sequence non-neural-network state-of-the-art error correction and constrained codes for DNA data storage.^13^ The results for the evaluations, including latent redundancy bits, indicate potential further improvements with longer training time, while a high number of constraint-free sequences can be generated by fine-tuning the model by using the stability score as an additional metric to train the encoder.

Further potential improvements in error correction and constraint adherence performance could be achieved by optimizing the hyperparameters for different encoder, transcoder, and decoder combinations. Additionally, multiple potential improvements to the basic TurboAE implementation are described, which could potentially be used to improve the performance of Autoturbo-DNA, for example, utilizing a trainable interleaver,^22^^,^^23^ or more sophisticated training strategies.^24^ Our results indicate that neural-network-based codecs could be a viable alternative to traditional codecs for the DNA data storage channel.

### Limitations of the study

While close to state-of-the-art performance of non-neural network based codes, under the tested conditions and hyperparameters, Autoturbo-DNA does not yet surpass the error correction performance of traditionally designed codes. Furthermore, it cannot guarantee adherence to constraints.

## STAR★Methods

### Key resources table

**Table:** 

REAGENT or RESOURCE	SOURCE	IDENTIFIER
**Deposited data**		

Autoturbo-DNA	This study	Zenodo: doi:10.5281/zenodo.8385324

**Software and algorithms**		

Python 3.7, 3.11	python.org	N/A
PyTorch	pytorch.org	N/A
NumPy	numpy.org	N/A
Pandas	pandas.pydata.org	N/A
Matplotlib	matplotlib.org	N/A
Seaborn	seaborn.pydata.org	N/A
Statsmodels	statsmodels.org	N/A
SciPy	Scipy.org	N/A
PyTorch-Ignite	pytorch-ignite.ai	N/A
regex	pypi.org/project/regex/	N/A

### Resource availability

#### Lead contact

Further information and requests for resources should be directed to and will be fulfilled by the lead contact, Dominik Heider (dominik.heider@uni-marburg.de).

#### Materials availability

This study did not generate new materials.

#### Data and code availability

• All data reported in this paper will be shared by the lead contact upon request.
• All original code has been deposited at GitHub: https://github.com/MW55/autoturbo_dna ^25^ and Zenodo: https://doi.org/10.5281/zenodo.8385324. The code is publicly available under the MIT license as of the date of publication. DOIs are listed in the key resources table.
• Any additional information required to reanalyze the data reported in this paper is available from the lead contact upon request.

### Method details

#### Channel design

To train a model that can effectively repair erroneous sequences, the channel has to be modeled as close to the real DNA channel as possible. Autoturbo-DNA supports many different DNA data storage channel components, as shown in Table S5. The supported error rates and patterns are based on literature data collected by.^15^ Given the rapid development in DNA synthesis and sequencing, together with potential alternative storage media, like silica particles,^26^ error rates and -patterns need to be highly customizable. Autoturbo-DNA allows for such customization by utilizing JSON files for the channel parameters. The JSON files are cross-compatible with the configuration files used in the error simulator MESA.^15^ This interoperability between Autoturbo-DNA and MESA allows users to use the MESA error probability customization GUI to generate complex error patterns using a simple-to-use, click-and-drag-based interface. An example of how to generate a JSON configuration file for the Autoturbo-DNA channel simulator using the MESA GUI is shown in Figure S1.

#### Framework structure

All methods, optional components, and Autoturbo-DNA hyperparameters are embedded into a PyTorch^27^ framework. Each option can be selected and changed using command line arguments or by supplying a configuration file. The options supported by Autoturbo-DNA are described in Tables S1–S4 Error sources and their properties, as well as error probabilities for breaches of constraints, can be supplied in JSON files. After each epoch, the current optimizer state, model, configuration file, and a log file containing the loss value of each component, as well as the accuracy, stability, noise level, and percentage of correctly recovered blocks, are saved. The training can be paused anytime and will resume after the last evaluated epoch. Hyperparameters not integral to the model structure, like the model type, number of layers, or activation functions, can be adjusted during training, allowing online hyperparameter tuning.

#### Autoencoder structure

##### Interleaver

The codec combines three main components, as shown in Figure 1. Multiple options are available for each component, as shown in Tables S2–S4. One of the fundamental parts of turbo codes, and, by extension, turbo autoencoders, is the interleaver. The interleaver permutes the input sequence using either a pseudo-random function, with the seed of the function known to both the encoder and the decoder, or a deterministic interleaver with the general form^28^ of

(Equation 3) π(n)=kn+umodN,0≤n≤N

with *k* and *n* as fixed integers, and *k* being relatively prime to *n*. In a traditional turbo code design, the interleaver serves to spread out burst errors, improving the convergence of the decoding algorithm.^28^ For turbo autoencoders, which are primarily evaluated using i.i.d channels, the interleaver adds long-range memory to the code structure instead of increasing its robustness against burst errors.^16^ For the DNA data storage channel, both the addition of long-range memory and the increased robustness of the code against burst errors by the application of an interleaver is of importance, as indel errors, which affect all bases following the error, can be interpreted as burst errors.

##### Encoder

Each of the supported encoder network structures shown in Table S2 supports a base code rate of either $\frac{1}{2}$ by encoding one copy of the input data without interleaving and one copy that is interleaved before being encoded, or $\frac{1}{3}$, in which two copies of the input data are first encoded separately without interleaving. One copy is interleaved before the encoding. The encoders further support more granular adjustments to the code rate by increasing neurons in the output layer. The basic structure of the encoder is shown in Figure 2. The output is further normalized as described in:^16^^,^^29^

(Equation 4) $$x_{i}=\frac{b_{i}-\mu \left(b\right)}{\sigma \left(b\right)}$$

with μ(b) and σ(b), respectively, being the mean and standard deviation of the block. As the output of the encoder has to be mapped to the four DNA nucleotides, $x^{DNA}\subseteq \left\{A,T,C,G\right\}$, the output of the encoder is binarized and combined with a straight-through estimator.^16^ Each separately encoded copy of the input data is, after encoding, concatenated with each other. A mapping function λ maps each bit pair to a base, with 0∧0=A, 0∧1=G,1∧0=T, and 1∧1=C. The encoder is trained, either separately or in conjunction with the other parts of the codec, using the smooth L1 loss^30^^,^^31^ between the input data and the output of the decoder:

(Equation 5) $$L\left(u,\hat{u}\right)=L={\left\{l_{1},\ldots ,l_{N}\right\}}^{T}$$

For a batch size of *N*, with

(Equation 6) $$l_{n}=\left\{ \begin{array}{cc} \frac{0.5{\left(u_{n}-{\hat{u}}_{n}\right)}^{2}}{\beta } & \text{if}\;\left|u_{n}-{\hat{u}}_{n}\right|<\beta \\ \left|u_{n}-{\hat{u}}_{n}\right|-0.5\beta & \text{otherwise} \end{array}\right.$$

The hyperparameter β is user-definable, with a default value 1.0. By encoding a message *u* into a higher dimensional representation *x*, guided by a loss function that takes into account the discrepancy between the input message *u* and the decoded message $\hat{u}$, the encoder learns to represent a given, arbitrary binary input sequence into a unique output sequence that is robust against errors. If the additional loss term $w^{DNA}$ is included during the training, the encoder further learns to encode the input data into a constraint-adhering representation.

##### Constraint adherence training

Additionally to training the encoder for error correction performance, it can also be trained to encode the data into a constraint-adhering representation. Autoturbo-DNA supports the constraints of GC content, homopolymer length, k-mer occurrence, and undesired motifs. The undesired motifs are supplied using a JSON file that contains for each undesired motif an entry consisting of the motif itself, the error probability that is associated with the occurrence of the motif, and, optionally, a description of the motif. The GC content, homopolymer length, and k-mer occurrence are defined in a JSON file as a graph’s x and y coordinates. For example, a GC content that has a 100% error probability between 0% and 40% GC content and between 60% and 100%, with the area between 40% and 60% having a 0% error probability, can be described with the points X = 0, Y = 100; X = 40, Y = 100; X = 41, Y = 0; X = 59, Y = 0; X = 60, Y = 100, and X = 100, Y = 100. This approach further allows interpolation to smooth curves between points. Besides manual editing, the JSON file, error probability plots for the GC content, homopolymer length, and k-mer occurrence can be created using the graphical interface of MESA, as Autoturbo-DNA is cross-compatible with the configuration files of MESA. This cross-compatibility allows the generation of complex patterns by dragging points of a graph or simply combining common undesired motifs from a large selection of pre-existing ones in MESA.

##### Indel reduction component

Upstream of the decoder D(·) is the indel reduction component Q(·). As the decoder requires a fixed-size input, the primary purpose of this component is to transform the noisy sequences into a fixed-size representation that can be used as input for the decoder. It further serves as a transcoder that maps the quaternary DNA sequences to either binary sequences or sequences of continuous values. Autoturbo-DNA supports a multitude of strategies for indel reduction, as shown in Table S3. By default, it is trained using the smooth L1 loss of the encoder output with the output of Q(·). The output of Q(·) is binarized to allow for this training. Alternatively, it is also possible to train the indel reduction component using the smooth L1 loss between the input and decoded data. This approach does not require binarization, and the continuous output of Q(·) can be passed to the decoder.

##### Decoder

The decoder component follows the principles established by.^16^ It comprises two decoders, concatenated in serial, that iteratively update their prediction by utilizing the posterior of the previous decoder as prior. For a given input sequence, the sequence is split into multiple sub-sequences corresponding to the encoder’s two or three output sequences. The amount of sequences the input is split into depends on the chosen rate, with two sequences for a rate of $\frac{1}{2}$ and three sequences for a rate of $\frac{1}{3}$. For a rate of $\frac{1}{3}$, $y_{0}$ and $y_{1}$ are used as input to the first decoder, with a prior initialized as a tensor consisting only of zeros, the same size as the input subsequences. The output of the first decoder is then transposed, using the interleaving function with the same seed as used by the encoder, leading to the same interleaving pattern. Besides the posterior of the first decoder, the second decoder also takes as input $y_{2}$, corresponding to the interleaved encoded subsequence, and $y_{0}$, interleaved using the same seed as the encoder. The inverse function of the interleaver is then applied to the posterior of the second decoder, which is subsequently passed to the first decoder as prior. This sequence is repeated until a user-definable amount of iterations is reached. The output of the final iteration is then passed to a sigmoid activation function, resulting in the final output sequence. A schematic of the decoding process for a rate of $\frac{1}{3}$ is shown in Figure 3. The input of the first decoder is y0 and the deinterleaved $y_{1}$, together with the prior, as described above. In contrast, the input for the second decoder is the interleaved form of $y_{0}$, together with $y_{1}$ and the interleaved prior. The structure of two decoder blocks, forming a connection by utilization of the output of one decoder block as additional input for the other decoding block, together with the discrepancy between input message *u* and decoded message $\hat{u}$ as loss metric, allows the decoder to learn to iteratively improve predictions regarding the input message that is reminiscent of the principles established with the development of turbo codes. Further, given the quality variance of DNA sequencing outputs,^32^ the approach of utilizing multiple, separately encoded copies of a sequence as input for the decoder is well suited for the DNA data storage channel.

### Quantification and statistical analysis

Data analysis was performed using Python 3.3.7, Pandas 1.2.4, Matplotlib 3.4.2, Seaborn 0.11.2, Numpy 1.19.5, Statsmodels 0.12.2, and SciPy 0.12.2. The boxplots of Figure S2 consist of the different combinations of four encoder architectures, four decoder architectures, and three transcoder architectures. The mean and median of the boxplots represent the mean reconstruction accuracy and median reconstruction accuracy for the evaluated model groups after 400 epochs of training with random data.

Tests for statistical significance between groups of trained models were carried out using independent t-tests. Each group consists of six models, which are described in the legends of Figures 7, S4, and S6. Tests for normality were carried out using the Shapiro-Wilk test, and tests for the homogeneity of variances were carried out using Levene’s test.

For the reconstruction accuracy, both before and after fine-tuning, the Shapiro-Wilk test yielded W = 0.926 with a *p*-value of 0.336 (after fine-tuning) and W = 0.943 with a *p*-value of 0.534 (before fine-tuning). Correspondingly, Levene’s test indicated a statistic of 0.097 and a *p*-value of 0.758, suggesting homogeneity of variances between these groups.

Regarding the stability score, the Shapiro-Wilk test results were W = 0.929 with a *p*-value of 0.372 (after fine-tuning) and W = 0.974 with a *p*-value of 0.95 (before fine-tuning). Levene’s test for these groups showed a statistic of 0.811 and a *p*-value of 0.378.

In the comparison involving training with the stability score as an additional training metric versus training without it, the Shapiro-Wilk test for the stability score reported W = 0.934 and a *p*-value of 0.426 (with the additional loss metric), and W = 0.974 with a *p*-value of 0.95 (without the additional loss metric). The Levene’s test for this comparison yielded a statistic of 0.0004 with a *p*-value of 0.985. For reconstruction accuracy in this context, the Shapiro-Wilk results were W = 0.893, *p*-value = 0.129 (with stability score) and W = 0.943, *p*-value = 0.534 (without stability score), with Levene’s test showing a statistic of 0.225 and a *p*-value of 0.64. The reults suggest that the data adhered to the assumptions of normality and homogeneity of variances.
