# Supplementary material for: Turbo autoencoders for the DNA data storage channel with Autoturbo-DNA
Source: iScience. 2024 Mar 27;27(5):109575. doi: 10.1016/j.isci.2024.109575 (PMC11024904; doi:10.1016/j.isci.2024.109575)
Supplement: Document S1. Figures S1–S6 and Tables S1–S8 [file mmc1.pdf]

iScience, Volume *27*

Supplemental information

Turbo autoencoders for the DNA data storage channel with Autoturbo-DNA

Marius Welzel, Hagen Dreßler, and Dominik Heider

1 Configuration parameters

Parameters

Option Strings	Type	Default	Help
-h, –help	None	==SUPPRESS==	show this help message and exit.
-v, –version	None	==SUPPRESS==	show program's version number and exit.
–wdir	str	None	Path to the working directory, if not existing the model will be saved here, if already existing the model will be loaded.
–train	bool	False	Create and train the desired model.
–bitenc	None	None	Encode with a model a bit string into a code.
–bitdec	None	None	Decode with a model a bit string into a code.
–encode, -e	None	None	Encode with a model a file.
–decode, -d	None	None	Decode with a model a code back into a file.
–input, -i	str	None	Path to the file to be en-/decoded.
–output, -o	str	None	Path to the output file.
–index_size, -is	int	16	size (in bits) of the added index, larger files need bigger index sizes, has to be a multiple of 8.
–simulate	None	None	Simulate errors on a generated code.
–ids	None	False	Shows a list of the default ids of the different options for DNA synthesis, storage and sequencing simulation.
–seed	int	0	Specify a integer number, this allows to reproduce the results.
–gpu	None	False	Whether the calculations of the models should run on the GPU (using CUDA).
–parallel	None	False	Whether to run the calculations on multiple GPUs, if there are more than one.
–threads	int	8	If using the CPU, how many threads should be used.
–rate	str	onethird	Rate of the code, supported are 1/3 (argument=onethird) and 1/2 (argument=onehalf).
–block-length	int	64	Length of the bitstreams to be used
–block-padding	int	18	Length of the padding by which the bitstream is extended.
–encoder	str	cnn	Choose which encoder to use
–enc-units	int	64	The number of expected features in the hidden layer for the encoder.
–enc-actf	str	elu	Choose which activation function should be applied to the encoder: tanh, elu, relu, selu, sigmoid or identity.
–enc-dropout	float	0.0	Dropout probability for the encoder.
–enc-layers	int	5	Number of recurrent layers per RNN/CNN structure in the encoder.
–enc-kernel	int	5	Size of the kernels for the CNN in the encoder
–enc-rnn	str	GRU	Choose which structure to use for the RNN in the encoder: GRU or LSTM.
–vae-beta	float	0.0	The beta multiplier of the Kullback–Leibler divergence if using a VAE.
–decoder	str	cnn	Choose which decoder to use.
–dec-units	int	64	The number of expected features in the hidden layer for the decoder.

Continued on next page

Option Strings	Type	Default	Help
–dec-actf	str	identity	Choose which activation function should be applied to the decoder: tanh, elu, relu, selu, sigmoid or identity.
–dec-dropout	float	0.0	Dropout probability for the decoder.
–dec-layers	int	5	Number of recurrent layers per RNN/CNN structure in the decoder.
–dec-inputs	int	5	The number of expected input features for the decoder.
–dec-iterations	int	6	Number of iterative loops to be made in the decoder.
–dec-kernel	int	5	Size of the kernels for the CNN in the decoder.
–dec-rnn	str	GRU	Choose which structure to use for the RNN in the decoder: GRU or LSTM.
–not-extrinsic	None	True	Whether extrinsic information should be applied to the decoder each iteration.
–coder	str	cnn	Choose which coder to use.
–coder-units	int	64	The number of expected features in the hidden layer for the coder.
–coder-actf	str	elu	Choose which activation function should be applied to the coder: tanh, elu, relu, selu, sigmoid or identity.
–coder-dropout	float	0.0	Dropout probability for the coder.
–coder-layers	int	5	Number of recurrent layers per RNN/CNN structure in the coder.
–coder-kernel	int	5	Size of the kernels for the CNN in the coder.
–coder-rnn	str	GRU	Choose which structure to use for the RNN in the coder: GRU or LSTM.
–init-weights	str	None	Choose which method to use to initialize the linear layers of the model: normal, uniform, constant, xavier_normal, xavier_uniform, kaiming_normal or kaiming_uniform.
–lat-redundancy	int	0	Redundancy of the final encoder layer (and first decoder layer), required to account for constraints. Has to be divisible by 2.
–ens-models	int	3	If ensemble coders are used, defines the number of coder instances in the ensemble.
–padding-style	str	constant	If padding should be constant values or a circular copy of the input.
–blocks	int	1024	Number of the bitstreams to be used.
–batch-size	int	256	Size of the batch to be used during training.
–epochs	int	100	Number of epochs the whole model should be trained.
–enc-lr	float	0.00001	Value of the learning rate to be used for the encoder.
–enc-optimizer	str	adam	Choose which optimizer to use for the encoder: Adam, SGD or Adagrad.
–enc-steps	int	1	Number of training steps to be performed per epoch for the encoder.
–dec-lr	float	0.00001	Value of the learning rate to be used for the decoder.
–dec-optimizer	str	adam	Choose which optimizer to use for the decoder: Adam, SGD or Adagrad.

Continued on next page

Option Strings	Type	Default	Help
–dec-steps	int	2	Number of training steps to be performed per epoch for the decoder.
–coder-lr	float	0.001	Value of the learning rate to be used for the coder.
–coder-optimizer	str	adam	Choose which optimizer to use for the coder: Adam, SGD or Adagrad.
–coder-steps	int	5	Number of training steps to be performed per epoch for the coder.
–simultaneously	None	False	Whether the encoder and decoder are to be trained at the same time, if so, the learning parameters from the encoder are used.
–batch-norm	bool	False	Whether to use batch normalization or not.
–separate-coder-training	None	False	If the coder should be split into 3 seperate instances during training.
–all-errors	None	False	train each part of the model always with all error types.
–channel	str	dna	which channel model should be used for training.
–continuous-coder	None	False	toggles that the intermediate decoder (coder) passes continuous values to the decoder.
–constraint-training	None	False	If the code should also be trained to adhere to constraints.
–loss-beta	float	1.0	beta parameter for the smooth L1 loss.
–coder-train-target	str	encoded_data	how the coder should be trained, for best reconstruction accuracy or to be as close to the encoder output as possible.
–simultaneously-warmup	int	0	if using simultaneously training, how many warmup epochs should be trained seperatly, before moving to simultaneously training.
–synthesis	None	(1, None)	Specify the id of the synthesis method.
–pcr-cycles	int	30	Number of cycles to be used for the PCR.
–pcr	None	(14, None)	Specify the id of the PCR type.
–storage-months	int	24	Months of storage to be simulated.
–storage	None	(1, None)	Specify the id of the storage host.
–sequencing	None	(2, None)	Specify the id of the sequencing method.
–amplifier	float	5.0	Value by how much more distinct the errors should be.
–probabilities	str	probabilities.json	Path to json file for error probabilities.
–useq	str	undesired_sequences.json	Path to json file for undesired sequences.
–gc-window	int	50	Size of the window to be used for the GC-Content error probability detection.
–kmer-window	int	10	Size of the window to be used for the Kmer error probability detection.

Table 1: Supported parameters of Autoturbo-DNA, related to Figure 1.

Encoder

Parameter	Description
rnn	Recurrent neural network, each copy of the input is encoded by seperate RNNs.
srnn	Recurrent neural network, returns the unencoded inputs and 1 or 2 encoded copies, depending on the rate parameter.
cnn	Convolutional neural network, each copy of the input is encoded by seperate CNNs.
scnn	Convolutional neural network, returns the unencoded inputs and 1 or 2 encoded copies, depending on the rate parameter.
transformer	Encoding structure based on a transformer encoder. Each copy of the input is encoded by seperate instances.
vae	Variational autoencoder structure. Using CNN as base neural network.
cnn_kernel_inc	CNN with an increasing kernel size, first pass is through a CNN with half the kernel size of the parameter value, second is with the full kernel size.
resnet1d	One dimensional residual neural net.

Table 2: Implemented encoder structures, related to Figure 2.

Transcoder

Parameter	Description
mlp	Multilayer perceptron, the input is split into subsequences that correspond to the number of encoded copies and transcoded by seperate MLPs.
cnn	Convolutional neural network. Each subsequence is encoded by seperate instances.
rnn	Recurrent neural network. Each subsequence is encoded by seperate instances.
transformer	Transcoding structure based on a transformer encoder. Each subsequence is encoded by seperate instances.
cnn_rnn	A combination of CNNs and RNNs. Each subsequence is encoded by seperate instances.
cnn_conc	A single CNN that transcodes the input sequence without splitting it into subsequences.
cnn_ensemble	An ensemble of CNNs, using majority voting to return the most likely candidate sequence. Only useable with binary outputs.
resnet	Residual neural network, the inputs are transcoded by a one dimensional ResNet together and split afterwards, to be seperatly transcoded by a linear layer.
resnet2d	Residual neural network, the inputs are transcoded by a two dimensional ResNet together and split afterwards, to be seperatly transcoded by a linear layer. Before the transcoding, the interleaved sequence is deinterleaved.
resnet2d_1d	Residual neural network for rate 1/3 only. The not interleaved inputs are transcoded by a two dimensional ResNet together, while the interleaved input is seperatly transcoded by a one dimensional ResNet.
resnet_ens	An ensemble of the basic ResNet component, using majority voting to return the most likely candidate sequence. Only useable with binary outputs.
resnet_sep	ResNet components that are inpendently trained from each other for each subsequence.
resnet_conc	A single, one dimensional ResNet that transcodes the input sequence without splitting it into subsequences.
cnn_sep	CNNs that are inpendently trained from each other for each subsequence.

Table 3: Implemented indel reduction transcoders, related to Figure 1.

**Decoder**	
Parameter	Description
rnn	Recurrent neural network based decoder structure.
cnn	Convolutional neural network based decoder structure.
entransformer	Decoder structure based on a transformer encoder.
ensemble_dec	An ensemble of CNNs, using majority voting to return the most likely candidate sequence.
resnet1d	One dimensional residual neural net.

Table 4: Implemented decoder structures, related to Figure 3.

**Error rates**					
**Synthesis method**	**Error-correction**	**Deletion**	**Insertion**	**Substitution**	**Raw rate**
Column synthesized oligos	ErrASE	0.6	0.2	0.2	$2.50 \cdot 10^{-5}$
Microarray based oligo pools	ErrASE	0.6	0.2	0.2	$1.20 \cdot 10^{-3}$
Column synthesized oligos	MutS	0.7	0.15	0.15	$1.00 \cdot 10^{-4}$
Column synthesized oligos	Consensus Shuffle	0.7	0.15	0.15	$1.25 \cdot 10^{-4}$
**PCR Polymerase**	**Proofread**				
Taq	No	0.01	0	0.99	$4.30 \cdot 10^{-5}$
Pwo	Yes	0	0	1	$2.40 \cdot 10^{-6}$
Pfu	Yes	0	0	1	$2.80 \cdot 10^{-6}$
**Storage Host**	**Domain**				
E. coli	Prokaryotes	0.08	0.08	0.84	$3.17 \cdot 10^{-7}$
H. sapiens	Eukaryotes	0.06	0.06	0.88	$6.90 \cdot 10^{-11}$
M. musculus	Eukaryotes	0.025	0.025	0.95	$4.40 \cdot 10^{-9}$
D. melanogaster	Eukaryotes	0.33	0.33	0.34	$2.10 \cdot 10^{-8}$
S. cerevisiae	Eukaryotes	0.13	0.13	0.74	$7.90 \cdot 10^{-8}$
**Sequencing method**	**Submethod**				
Illumina	Single end	0.0024	0.0013	0.9963	$2.10 \cdot 10^{-3}$
Illumina	Paired end	0.0018	0.0011	0.79	$3.20 \cdot 10^{-3}$
Nanopore	1D	0.37	0.15	0.48	$2.00 \cdot 10^{-1}$
Nanopore	2D	0.36	0.23	0.41	$1.30 \cdot 10^{-1}$
PacBio	Subread	0.2	0.05	0.75	$2.00 \cdot 10^{-1}$
PacBio	CCS	0.21	0.42	0.37	$1.40 \cdot 10^{-1}$

Table 5: Out-of-the-box supported error rates for the channel simulation, related to Figure 1

**Used Hyperparameters**	
**Parameter**	**Value**
Amplifier	15
Epochs	400
Encoder dropout	0.2
Encoder layers	7
Encoder learning rate	0.00001
Encoder steps	20
Decoder layers	7
Decoder learning rate	0.00001
Decoder steps	30
Decoder iterations	10
Transcoder layers	7
Transcoder learning rate	0.00001
Transcoder steps	20
Transcoder-units	128
Transcoder train target	Encoded data
Combined steps	20
Weight initialization	Normal
Block length	8

Table 6: Hyperparameters used in the evaluations that deviate from the default Autoturbo-DNA hyperparameters, related to Figure 4 to 7

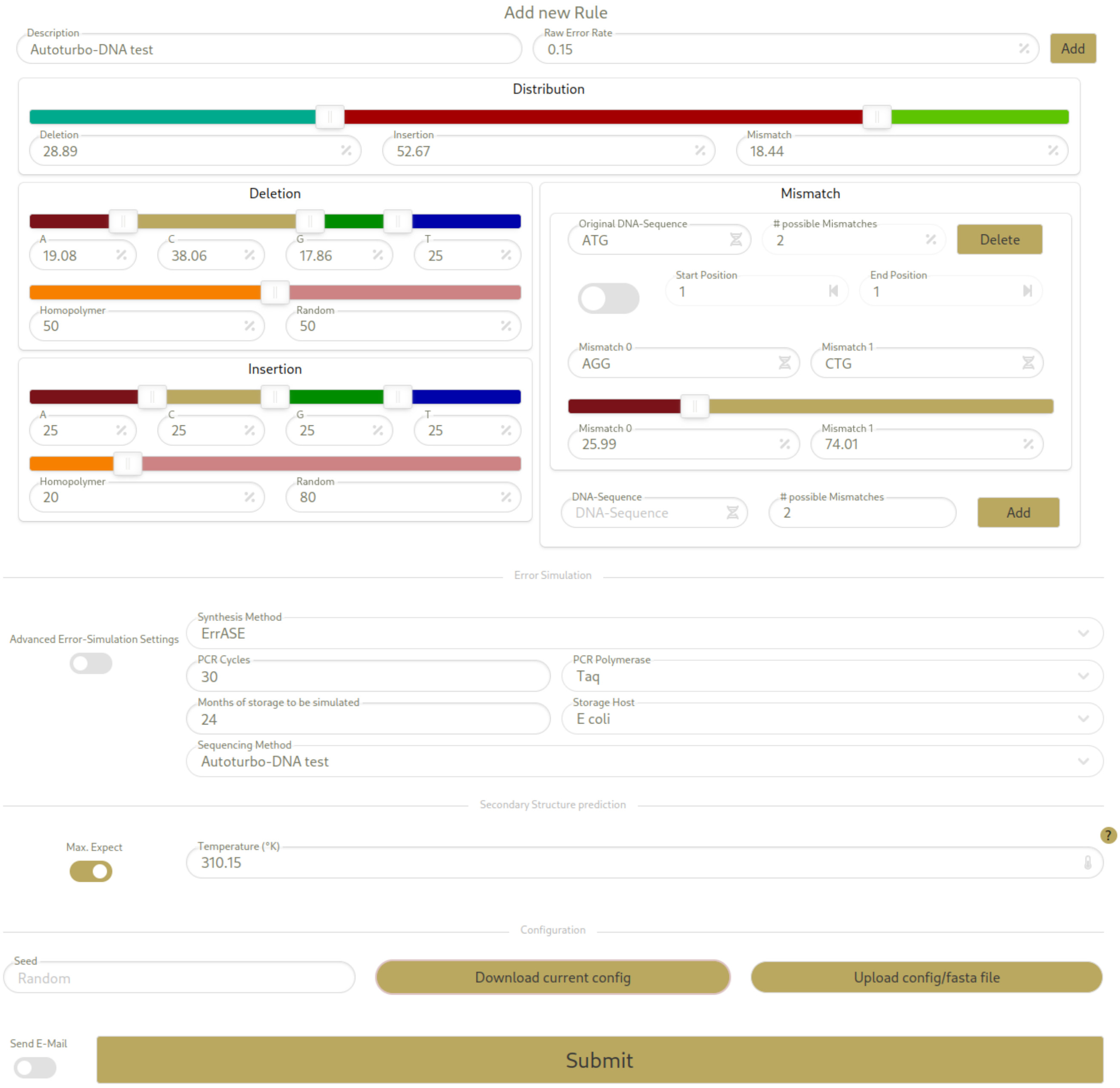

Figure 1: Example of generating a configuration file that can be used to train models with Autoturbo-DNA, related to Figure 1. Top: the MESA interface to design a new rule, showing the ability to name the rule (here "Autoturbo-DNA test"), defining the raw error rate, the distribution of errors between deletions, insertions, and substitutions/mismatches, and the distribution and positions for each error type. For example, an insertion happens to 80 % at a random position and 20 % in a homopolymer, and the inserted base is one of the four bases with equal probability. Bottom: the simulation interface of MESA, with the new rule chosen as the sequencing method. Close to the bottom of the image, the "Download current config" button allows the download of a JSON file containing all parameters. The JSON file data can then be used for training by adding it to the corresponding config/error_sources file of Autoturbo-DNA. The new error profile can then be selected by its ID using the related hyperparameter (either –sequencing, –synthesis, –pcr, or –storage).

3 · 8 block size slopes

Model	Slope
cnn_cnn_resnet_2_8	$1.412 \cdot 10^{-6}$
cnn_cnn_resnet_4_8	$1.212 \cdot 10^{-7}$
cnn_cnn_resnet_8_8	$2.170 \cdot 10^{-7}$
vae_cnn_resnet_2_8	$1.966 \cdot 10^{-6}$
vae_cnn_resnet_4_8	$1.246 \cdot 10^{-6}$
vae_cnn_resnet_8_8	$9.408 \cdot 10^{-7}$

Table 7: Slopes of the last 200 epochs for the evaluated models using a block size of $3 \cdot 8$, related to Figure 5. The naming convention is encoder, decoder, transcoder, latent redundancy, block length.

3 · 18 block size slopes

Model	Slope
cnn_cnn_resnet_2_16	$1.956 \cdot 10^{-6}$
cnn_cnn_resnet_4_16	$-2.422 \cdot 10^{-7}$
cnn_cnn_resnet_8_16	$4.868 \cdot 10^{-6}$
vae_cnn_resnet_2_16	$2.878 \cdot 10^{-6}$
vae_cnn_resnet_4_16	$2.377 \cdot 10^{-6}$
vae_cnn_resnet_8_16	$2.944 \cdot 10^{-6}$

Table 8: Slopes of the last 200 epochs for the evaluated models using a block size of $3 \cdot 16$, related to Figure 6. The naming convention is encoder, decoder, transcoder, latent redundancy, block length.

2 Results

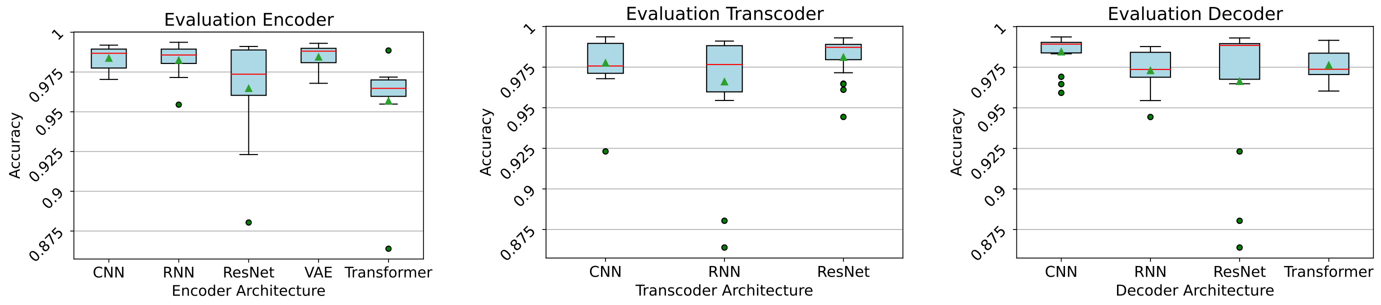

Figure 2: Boxplot of the accuracy of the trained models, separated either by the used encoder, transcoder or decoder architecture, related to Figure 1 to 3. A red line represents the median, a green triangle represents the mean, and the outliers are represented by green dots.

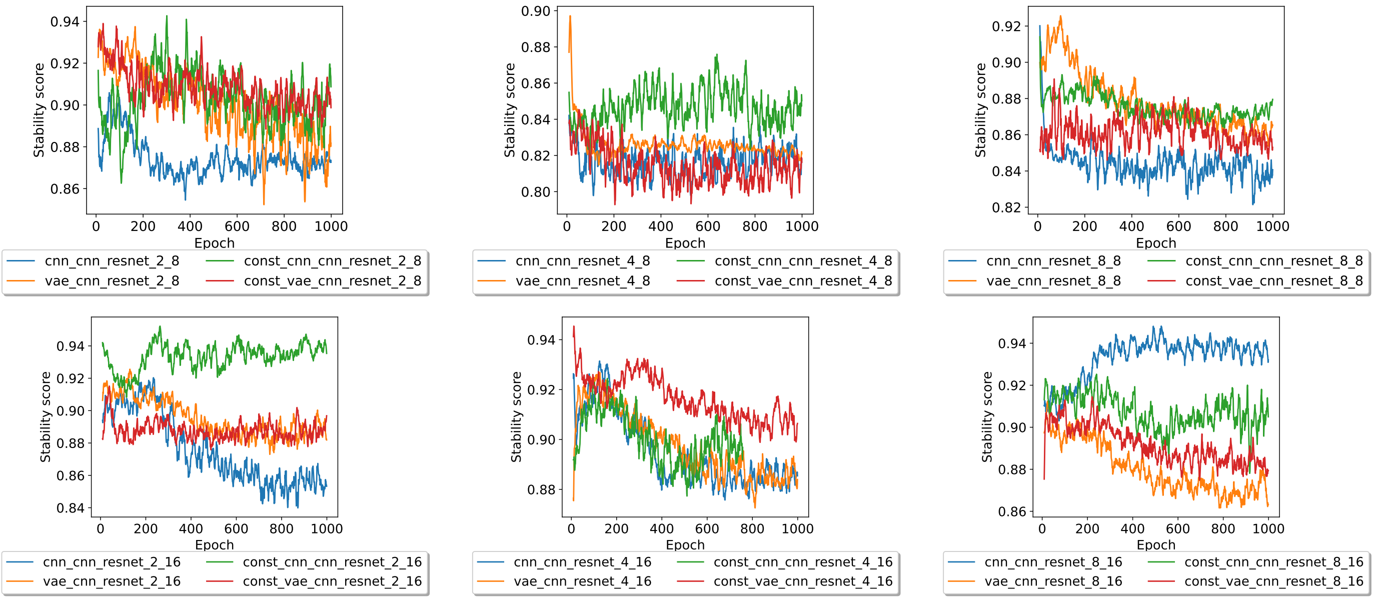

Figure 3: Stability score over 1000 epochs in a 10 epoch rolling average for a latent redundancy of 2 bits (left), 4 bits (middle) and 8 bits (right), related to Figure 7. On the top, the models were trained using a block size of $3 \cdot 8$, and on the bottom, the training was carried out with a block size of $3 \cdot 16$. The legend labels are structured in the form of constraint adherence training, encoder, decoder, transcoder, latent redundancy, block size.

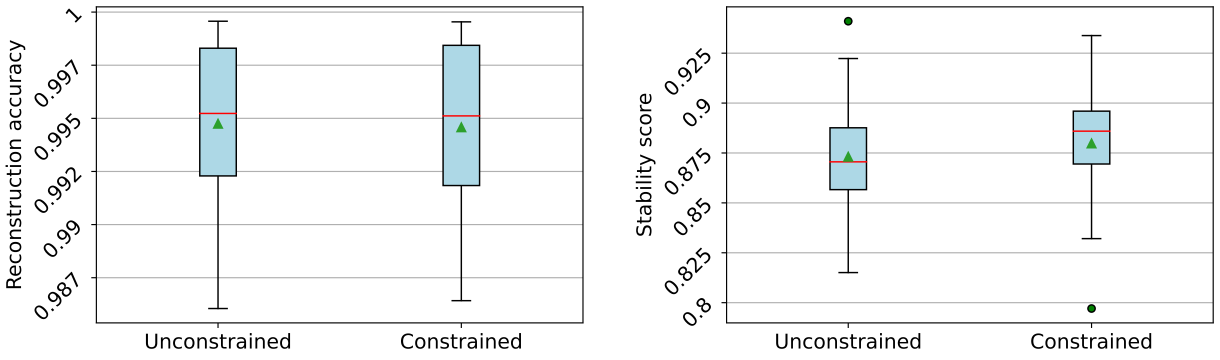

Figure 4: Boxplots of the reconstruction accuracy score (left) and the stability score (right) of models trained without (left) and with (right) the stability score as training metric, related to Figure 7. The models were further trained with either 2, 4, or 8 bits of latent redundancy and a block size of either $3 \cdot 8$ or $3 \cdot 16$ bits. A red line represents the median, a green triangle represents the mean, and the outlier are represented by green dots.

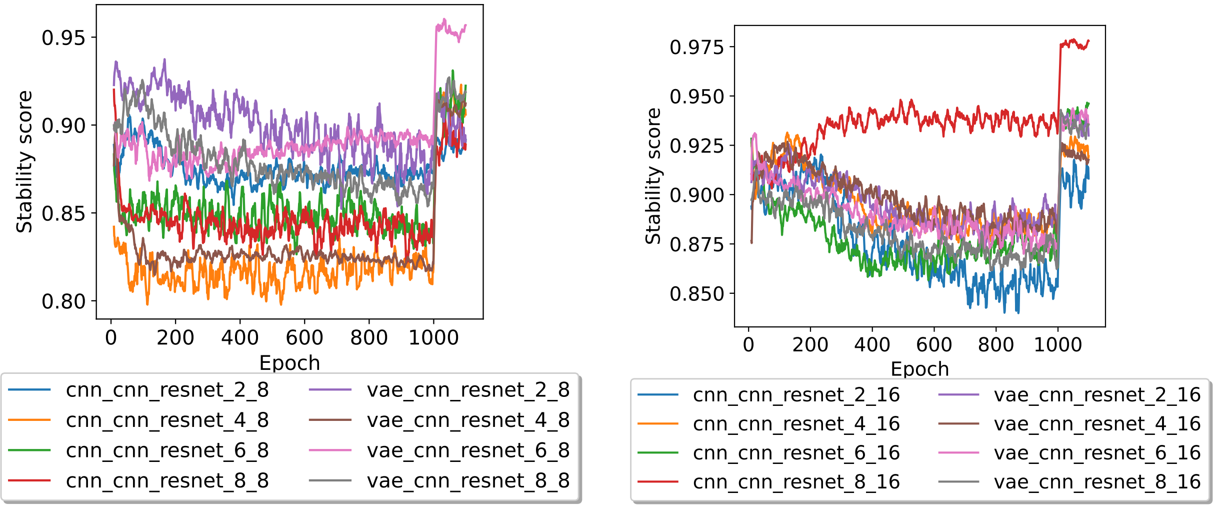

Figure 5: Stability score for different block lengths in a 10 epoch rolling average, for a block size of $3{\cdot}8$ (left) and $3{\cdot}16$ (right), related to Figure 7. Each model was trained for 1000 epochs without taking the stability score into account when training the encoder, followed by 100 epochs with the stability score being taking into account when training the encoder. The legend labels are structured in the form of encoder, decoder, transcoder, latent redundancy, block size.

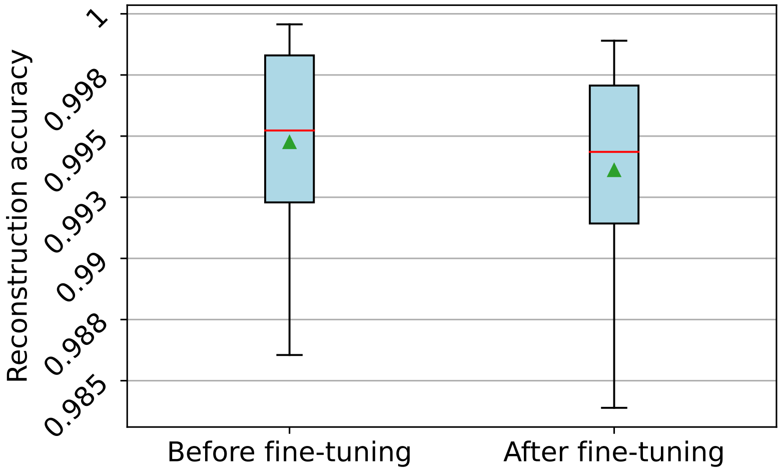

Figure 6: Boxplot of the reconstruction accuracy score of models trained before (left) and after (right) fine-tuning by utilizing the stability score as additional training metric, related to Figure 7. The models were further trained with either 2, 4, or 8 bits of latent redundancy and a block size of either $3 \cdot 8$ or $3 \cdot 16$ bits. A red line represents the median, a green triangle represents the mean, and the outlier are represented by green dots.
